# Global mapping of interventions to improve the quality of life of patients with cardiovascular diseases during 1990–2018

**DOI:** 10.1186/s12955-020-01507-9

**Published:** 2020-07-29

**Authors:** Bach Xuan Tran, Son Nghiem, Clifford Afoakwah, Giang Hai Ha, Linh Phuong Doan, Thao Phuong Nguyen, Tuan Thanh Le, Carl A. Latkin, Cyrus S. H. Ho, Roger C. M. Ho

**Affiliations:** 1 Department of Health Economics, Institute for Preventive Medicine and Public Health, Hanoi Medical University, No.1 Ton That Tung street, Dong Da, Hanoi, Vietnam; 2 Bloomberg School of Public Health, Johns Hopkins University, Baltimore, MD USA; 3 Centre for Applied Health Economics (CAHE), Griffith University, Brisbane, Australia; 4 Institute for Global Health Innovations, Duy Tan University, Da Nang, Vietnam; 5 Center of Excellence in Evidence-based Medicine, Nguyen Tat Thanh University, Ho Chi Minh City, Vietnam; 6 Center of Excellence in Behavioral Medicine, Nguyen Tat Thanh University, Ho Chi Minh City, Vietnam; 7 Echo-lab, Vietnam National Heart Institute, Bach Mai Hospital, Hanoi, Vietnam; 8 Department of Psychological Medicine, National University Hospital, Singapore, Singapore; 9 Department of Psychological Medicine, Yong Loo Lin School of Medicine, National University of Singapore, Singapore, Singapore; 10 Institute for Health Innovation and Technology (iHealthtech), National University of Singapore, Singapore, Singapore

**Keywords:** Scientometrics, Content analysis, Text mining, Interventions, CVD, QOL, Global, Mapping

## Abstract

**Background:**

Cardiovascular diseases (CVDs) have been the global health problems that cause a substantial burden for the patients and the society. Assessing the Quality of Life (QOL) of CVD patients is critical in the effectiveness evaluation of CVD treatments as well as in determining potential areas for enhancing health outcomes. Through the adoption of a combination of bibliometric approach and content analysis, publications trend and the common topics regarding interventions to improve QOL of CVD patients were searched and characterized to inform priority setting and policy development.

**Methods:**

Bibliographic data of publications published from 1990 to 2018 on interventions to improve QOL of CVD patients were retrieved from Web of Science. Network graphs illustrating the terms co-occurrence clusters were created by VOSviewer software. Latent Dirichlet Allocation approach was adopted to classify papers into major research topics.

**Results:**

A total of 6457 papers was analyzed. We found a substantial increase in the number of publications, citations, and the number of download times of papers in the last 5 years. There has been a rise in the number of papers related to intervention to increase quality of life among patients with CVD during 1990–2018. Conventional therapies (surgery and medication), and psychological, behavioral interventions were common research topics. Meanwhile, the number of papers evaluating economic effectiveness has not been as high as that of other topics.

**Conclusions:**

The research areas among the scientific studies emphasized the importance of interdisciplinary and inter-sectoral approaches in both evaluation and intervention. Future research should be a focus on economic evaluation of intervention as well as interventions to reduce mental issues among people with CVD.

## Background

Cardiovascular diseases (CVDs) have been the global health problem with rising prevalence, incidence, and death rates. In 2017, 31.8% (17.79 million cases) of global deaths were attributed to CVDs, plus nearly 336 million disability-adjusted life-years (DALY) [1]. Ischemic heart disease (IHD) and stroke account for the highest standardized death rate among CVD causes [2]. Episodes of CVDs have complex, long term impacts on the life of patients, which is far beyond survivorship, since their consequences cause impairments in physical and cognitive functioning, which, in turn, limit the daily activities and social interactions of the survivors [3–7].

Achieving good quality of life (QOL), therefore, is crucial to patients suffering from heart and stroke diseases [3, 8]. According to the definition of World Health Organization on QOL, QOL is “affected in a complex way by the person’s physical health, psychological state, personal beliefs, social relationships and their relationship to salient features of their environment” [9]. QOL can be considered as one of the most important outcomes in healthcare, particularly among patients with CVDs [10]. Deterioration of QOL among CVD patients is positively correlated with higher rates of hospital readmission and fatality [11, 12]. Hence, assessing the QOL of CVD patients is critical when evaluating the effectiveness of CVD treatments and determining which aspects should be improved.

There is a remarkable growth in the body of literature regarding manners to improve the QOL of people with heart and stroke diseases. These implications range from healthier lifestyle encouragement [13] to the incorporation of mental health treatments into routine CVDs management [6]. Moreover, some initiatives have been implemented comprising: adoption of collaborative care models [14]; support and counseling of CVD specialists in the primary care setting to people at-risk of CVDs [15]; or adoption of surgical procedures, for instance, coronary artery bypass graft surgery (CABG) instead of percutaneous coronary intervention (PCI) [16].

Several systematic reviews worked on interventions that could improve the QOL of people with heart and stroke diseases. For example, life-style interventions at the workplace could decrease the risk of CVD [17], or community-based nursing interventions increase the outcome of treatment for people with CVD [18]. Besides, Widmer et al. confirmed the effectiveness of digital health interventions for the prevention of cardiovascular disease [19]. Despite the abundance of documents on the QOL and interventions targeting people with heart and stroke diseases, there is a lack of publications offering a ‘big picture’ of the interaction between interventions and QOL among CVD patients. This limits the ability of healthcare providers and policymakers to identify pathways to efficiently allocate scarce resources in CVD treatment.

The bibliometric approach has been proposed to be a potential solution given the capacity to provide a comprehensive and holistic investigation of the literature. By combining bibliometric approach and content analysis, we aimed at providing an interdisciplinary insights into research areas as well as characterizing the most common topics regarding interventions to improve QOL of CVD patients. Our findings can inform priority setting and policy development towards sustainable efforts of enhancing the lives of people with these conditions.

## Methods

### Search strategy, keywords, data download and extraction

The published works regarding the QOL of CVD patients were downloaded from the Web of Science (WOS). For bibliometric analysis, the WOS is superior to Scopus or Medline/Pubmed because it: 1) allows to extract a large number of with full information (e.g titles, author names, total citation, total download times); 2) covers citation of scientific publications since 1900; and 3) comprises high impact scientific journals worldwide [20, 21]. Data were collected in March 2019; thus, we excluded the publications from 1st January 2019 onwards. The analysis focused on English articles and reviews, therefore, other document types such as letter to editors, or conference abstracts in any other languages were excluded. Two steps of the search strategy were performed as follow:

- Step 1: The terms “Quality of life”, and “well-being” were used to extract scientific research mentioning Quality of life on TS research in WOS (title, abstract, keywords, and topic) (see Additional file 1). Data were downloaded separately by two researchers and verified by a senior researcher. Any inconsistency between the data downloaded by the two researchers was solved by discussion and the data were re-downloaded when necessary. Papers which were 1) not articles and reviews; 2) not published in English; 3) written by anonymous authors. Final set of data was converted into txt format and transferred into STATA version 14.0 (STATACorp., Texas, USA) for further extraction and analysis.
- Step 2: A set of keywords related to CVDs was built, which based on the definition of World Health Organization (WHO), (“Coronary heart disease” OR “Heart failure” OR “Rheumatic Heart Disease” OR “Cerebrovascular disease”) [22], MeSH terms (“Cardiac arrhythmias” OR “Carcinoid heart disease” OR “Cardiac conduction system disease” OR “High cardiac output” OR “Low cardiac output” OR “Cardiomegaly” OR “Endocarditis” OR “heart aneurysm” OR “Heart arrest” OR “Congenital heart defects” OR “Heart neoplasms” OR “Heart rupture” OR “Heart attack” OR “Heart valve disease” OR “Myocardial ischemia” OR “Myocardial Ischemia” OR “Pericardial Effusion” OR “Pericarditis” OR “Ventricular Dysfunction” OR “Ventricular Outflow Obstruction” OR “Cardiovascular abnormalities” OR “Vascular malformations” OR “Cardiovascular infections”) [23], some systematic reviews (such as (“Ischemic heart disease” OR “Heart attack” OR “Stroke” OR “ischemic stroke” OR “Hemorrhagic stroke” OR “brain attack”) [24, 25]. Then, we used the terms “intervention*” or “trial*” to extract the papers regarding the intervention of this health problem (see Additional file 2).

### Data analysis

First, basic characteristics of publications were described, which included 1) years of publication; 2) the number of papers per year; 3) total citations of each year up to 2018 (from 1990 to 2018); 4) a total of download times (total usage) and average download times per year (mean usage per year) in the last 6 months; and 5) total of download times (total usage) and the average number of citation per year (mean citation rate per year) in the last 5 years. A network graph showing the co-occurrence of authors’ keywords was generated by the VOSviewer software tool (https://www.vosviewer.com/). The Latent Dirichlet Allocation (LDA), a generative statistical model, was used for classifying publications into topics [26–30]. The LDA approach was selected because of its ability to group and explain trends and patterns in text content. The techniques used for each type of type are presented in Table 1.

**Table 1:** Summary of analytical techniques for each data types

Type of data	Unit of analysis	Analytical methods	Presentations of results
Keywords	Words	Frequency of co-occurrence	1) Map of authors’ keywords
Abstracts	Papers	Latent Dirichlet Allocation	2) Ten classifications of research topics
WoS classification of research areas	WoS research areas	Frequency of co-occurrence	3) Dendrogram of research disciplines (WoS classification)

## Results

### Number of published items and publication trend

Table 2 reveals the characteristics of selected publications. The first paper was published in 1990. Then, there was a gradual increase in the number of interventions to improve QOL of patients with stroke and heart diseases during 1990–2018, resulting in a total of 6457 papers. Besides, the total number of download times (total usage), and the average number of download times (the mean use rate) in the last 5 years of papers published in 2013 were the highest compared with other years. Meanwhile, the total usage and the mean use rate last 6 months of the year 2019 were the highest figures compared with that of other years, which indicated the short-term interest of readers,

**Table 2:** General characteristics of publications

Year published	Total number of papers	Total citations	Mean cite rate per year	Total usage last 6 month	Total usage last 5 years	Mean use rate last 6 month	Mean use rate last 5 year
2018	700	737	1.05	1953	3045	2.79	0.87
2017	659	3666	2.78	989	4769	1.50	1.45
2016	578	6871	3.96	697	6667	1.21	2.31
2015	552	8684	3.93	533	6975	0.97	2.53
2014	464	9118	3.93	343	5737	0.74	2.47
2013	463	12,830	4.62	314	7335	0.68	3.17
2012	431	13,162	4.36	273	5818	0.63	2.70
2011	365	12,192	4.18	214	4187	0.59	2.29
2010	309	12,414	4.46	209	3095	0.68	2.00
2009	269	12,780	4.75	152	2357	0.57	1.75
2008	238	9916	3.79	125	1963	0.53	1.65
2007	241	14,878	5.14	109	1883	0.45	1.56
2006	184	12,221	5.11	71	1328	0.39	1.44
2005	182	14,436	5.67	64	1361	0.35	1.50
2004	159	15,142	6.35	118	1958	0.74	2.46
2003	119	9924	5.21	47	737	0.39	1.24
2002	103	14,757	8.43	48	958	0.47	1.86
2001	72	7578	5.85	30	493	0.42	1.37
2000	77	7457	5.10	17	397	0.22	1.03
1999	66	6225	4.72	24	421	0.36	1.28
1998	58	3524	2.89	14	221	0.24	0.76
1997	35	2735	3.55	12	229	0.34	1.31
1996	36	2995	3.62	6	104	0.17	0.58
1995	23	3295	5.97	3	147	0.13	1.28
1994	23	1451	2.52	5	70	0.22	0.61
1993	26	842	1.25	6	29	0.23	0.22
1992	13	599	1.71	0	31	0.00	0.48
1991	11	2880	9.35	6	71	0.55	1.29
1990	1	40	1.38	0	1	0.00	0.20

**Fig. 1:**
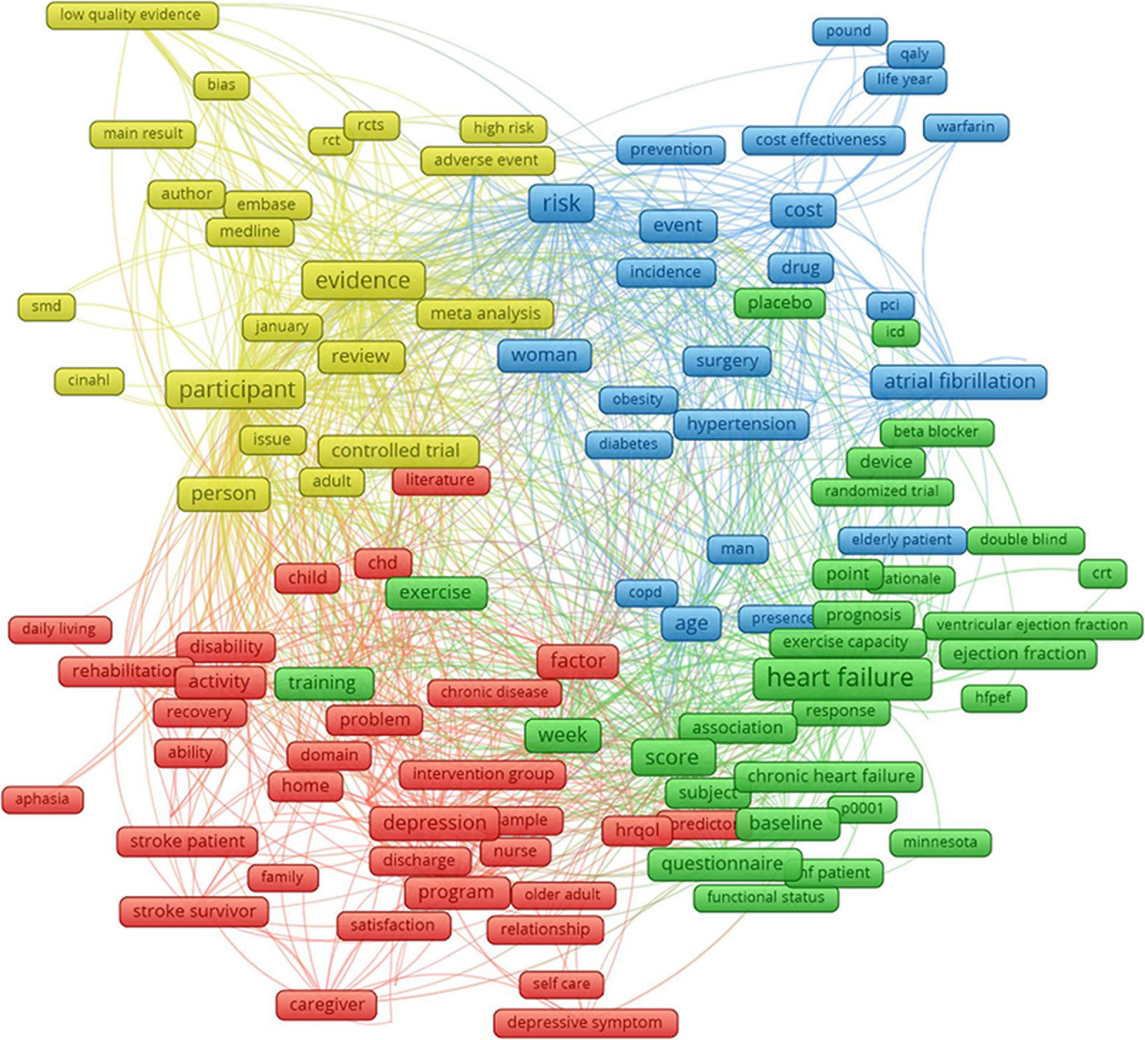
Co-occurrence of authors’ keywords. Note: the weight of a keyword determined its size of the label. The length of the lines shows the relatedness of keywords

The scope of studies was explored by using authors’ keywords, which was automatically mapped by the VOSviewer software tool. Figure 1 indicates four major clusters emerged from 164 most common keywords which appeared at least 200 times. Cluster 1 (red) refers to the quality of life, rehabilitation, and mental health illness of stroke patients. Cluster 2 (green) focuses on the daily exercise of patients with heart failure. Cluster 3 (yellow) includes types of analysis applied to identify the QOL among patients with heart diseases and stroke. Cluster 4 (blue) illustrates the risk factors, prevention, and cost-effectiveness of interventions for CVD.

Table 3 shows the most cited papers which had at least 400 citations. The title and abstract of each paper were reviewed by the research team and assigned to corresponding topics. Three major topics have been emerged encompassing: 1) Treatment of stroke or heart diseases (24/39 papers); 2) Preventions of stroke or heart diseases (8/39 papers), and 3) Others (systematic review and meta-analysis) (5/39 papers).

**Table 3:** Most cited papers

	Title	Journal	Cite	Year	Cite rate
1	Cardiac resynchronization in chronic heart failure	New England journal of medicine	3095	2002	182
2	Prevention of stroke by antihypertensive drug-treatment in older persons with isolated systolic hypertension - final results of the systolic hypertension in the elderly program (shep)	JAMA-journal of the American medical association	2233	1991	80
3	A randomized trial of the angiotensin-receptor blocker valsartan in chronic heart failure	New England journal of medicine	1887	2001	105
4	Advanced Heart Failure Treated with Continuous-Flow Left Ventricular Assist Device	New England journal of medicine	1625	2009	163
5	A multidisciplinary intervention to prevent the readmission of elderly patients with congestive-heart-failure	New England journal of medicine	1481	1995	62
6	Exercise-based rehabilitation for patients with coronary heart disease: Systematic review and meta-analysis of randomized controlled trials	American journal of medicine	1268	2004	85
7	Evidence suggesting that a chronic disease self-management program can improve health status while reducing hospitalization - A randomized trial	Medical care	1189	1999	59
8	Lifetime risk for development of atrial fibrillation - The Framingham Heart Study	Circulation	1100	2004	73
9	Combined cardiac resynchronization and implantable cardioversion defibrillation in advanced chronic heart failure - The MIRACLE ICD Trial	JAMA-journal of the American medical association	1026	2003	64
10	Effect of constraint-induced movement therapy on upper extremity function 3 to 9 months after stroke - The EXCITE randomized clinical trial	JAMA-journal of the American medical association	1022	2006	79
11	Treatment of heart failure guided by plasma aminoterminal brain natriuretic peptide (N-BNP) concentrations	Lancet	996	2000	52
12	Early decompressive surgery in malignant infarction of the middle cerebral artery: a pooled analysis of three randomised controlled trials	Lancet neurology	849	2007	71
13	Effects of controlled-release metoprolol on total mortality, hospitalizations, and well-being in patients with heart failure - The metoprolol CR/XL randomized intervention trial in congestive heart failure (MERIT-HF)	JAMA-journal of the American medical association	837	2000	44
14	Collaborative Care for Patients with Depression and Chronic Illnesses.	New England journal of medicine	767	2010	85
15	Evaluation study of congestive heart failure and pulmonary artery catheterization effectiveness - The ESCAPE trial	JAMA-journal of the American medical association	651	2005	47
16	Spironolactone for Heart Failure with Preserved Ejection Fraction	New England journal of medicine	618	2014	124
17	Continuous positive airway pressure for central sleep apnea and heart failure	New England journal of medicine	612	2005	44
18	Long-term benefits of biventricular pacing in congestive heart failure: Results from the multisite stimulation in cardiomyopathy (MUSTIC) study	Journal of the American college of cardiology	606	2002	36
19	Exercise training meta-analysis of trials in patients with chronic heart failure (extramatch)	BMJ-British medical journal	535	2004	36
20	Catheter ablation for atrial fibrillation in congestive heart failure	New England journal of medicine	533	2004	36
21	Does increased access to primary care reduce hospital readmissions?	New England journal of medicine	504	1996	22
22	Cardiac resynchronization and death from progressive heart failure - A meta-analysis of randomized controlled trials	JAMA-journal of the American medical association	501	2003	31
23	Effect of Phosphodiesterase-5 Inhibition on Exercise Capacity and Clinical Status in Heart Failure With Preserved Ejection Fraction A Randomized Clinical Trial	JAMA-journal of the American medical association	493	2013	82
24	Menopausal Hormone Therapy and Health Outcomes During the Intervention and Extended Poststopping Phases of the Women’s Health Initiative Randomized Trials	JAMA-journal of the American medical association	475	2013	79
25	Anger, anxiety, and depression as risk factors for cardiovascular disease: The problems and implications of overlapping affective dispositions	Psychological bulletin	460	2005	33
26	Cardiac resynchronization therapy for the treatment of heart failure in patients with intraventricular conduction delay and malignant ventricular tachyarrhythmias	Journal of the American college of cardiology	448	2003	28
27	The association of depression and anxiety with medical symptom burden in patients with chronic medical illness	General hospital psychiatry	441	2007	37
28	Combined intravenous and intra-arterial r-TPA versus intra-arterial therapy of acute ischemic stroke - Emergency management of stroke (EMS) bridging trial	Stroke	441	1999	22
29	Double-blind, placebo-controlled study of the effects of carvedilol in patients with moderate to severe heart failure - The PRECISE trial	Circulation	435	1996	19
30	Sex differences in stroke: epidemiology, clinical presentation, medical care, and outcomes	Lancet neurology	428	2008	39
31	A dose-dependent increase in mortality with vesnarinone among patients with severe heart failure	New England journal of medicine	425	1998	20
32	Surgical decompression for space-occupying cerebral infarction (the Hemicraniectomy After Middle Cerebral Artery infarction with Life-threatening Edema Trial [HAMLET]): a multi-centre, open, randomised trial	Lancet neurology	424	2009	42
33	Statins for the primary prevention of cardiovascular disease	Cochrane database of systematic reviews	423	2013	71
34	Effects of a multidisciplinary, home-based intervention on unplanned readmissions and survival among patients with chronic congestive heart failure: a randomised controlled study	Lancet	407	1999	20
35	Beraprost therapy for pulmonary arterial hypertension	Journal of the American college of cardiology	404	2003	25
36	Effect of Cinacalcet on Cardiovascular Disease in Patients Undergoing Dialysis	New England journal of medicine	403	2012	58
37	A randomized controlled trial of epoprostenol therapy for severe congestive heart failure: The Flolan International Randomized Survival Trial (FIRST)	American heart journal	400	1997	18

**Fig. 2:**
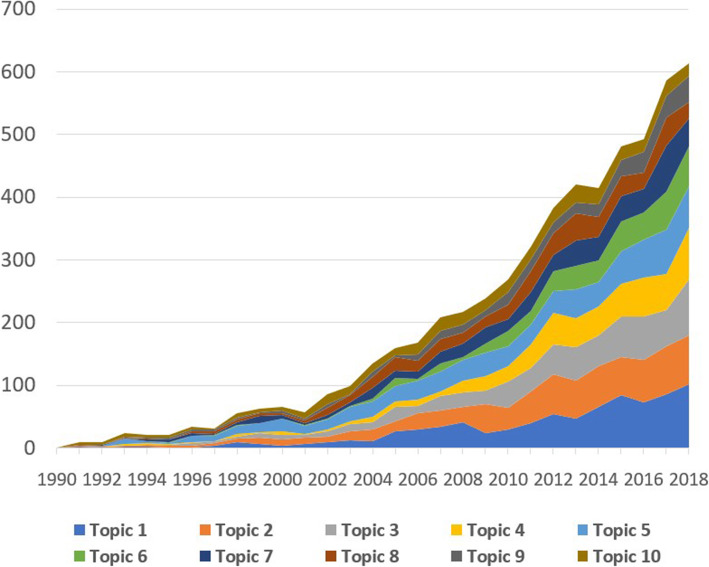
Changes in research topics development in QOL of CVD patients

The LDA was utilized to model the research topics based on texts in the abstracts and a total of ten major research topics was constructed (Table 4). Each topic was labeled by reviewing titles and abstracts of most cited papers within each group. Ten topics were divided into the following categories: 1) conventional therapies: topic 5, and topic 7; 2) other therapies: topic 2, topic 3, topic 4, topic 6, topic 8, topic 9, and topic 10. Figure 2 illustrates that recently, researchers have paid greater attention to two research topics focusing on biomedical therapies (topic 1), which are now under research and trial, as well as psychological and behavioral therapies for people with heart diseases (Topic 2).

**Table 4:** Ten research topics classified by LDA

Rank by the highest volume last 5 years	Research topics	N	Percent
Topic 1	Gene, Cell and Biomedical Therapies for heart diseases	799	14.1%
Topic 2	Psychological, Behavioral and Social impairments of patients with heart diseases	794	14.0%
Topic 3	Health Education, Motivation and Behavioral Interventions	670	11.8%
Topic 4	Technology-based, Robot-assisted Interventions on patients with Stroke	563	9.9%
Topic 5	Clinical, drug therapies for heart failure	751	13.2%
Topic 6	Traditional and Alternative Medicine for heart diseases	426	7.5%
Topic 7	Cardiac Surgeries and Interventions	506	8.9%
Topic 8	Economic Evaluation of Interventions on Patients with heart diseases and stroke	457	8.1%
Topic 9	Physical Activity and Exercise training for patients with heart failure	315	5.5%
Topic 10	Effectiveness of hormone replacement therapy on heart diseases	396	7.0%

**Fig. 3:**
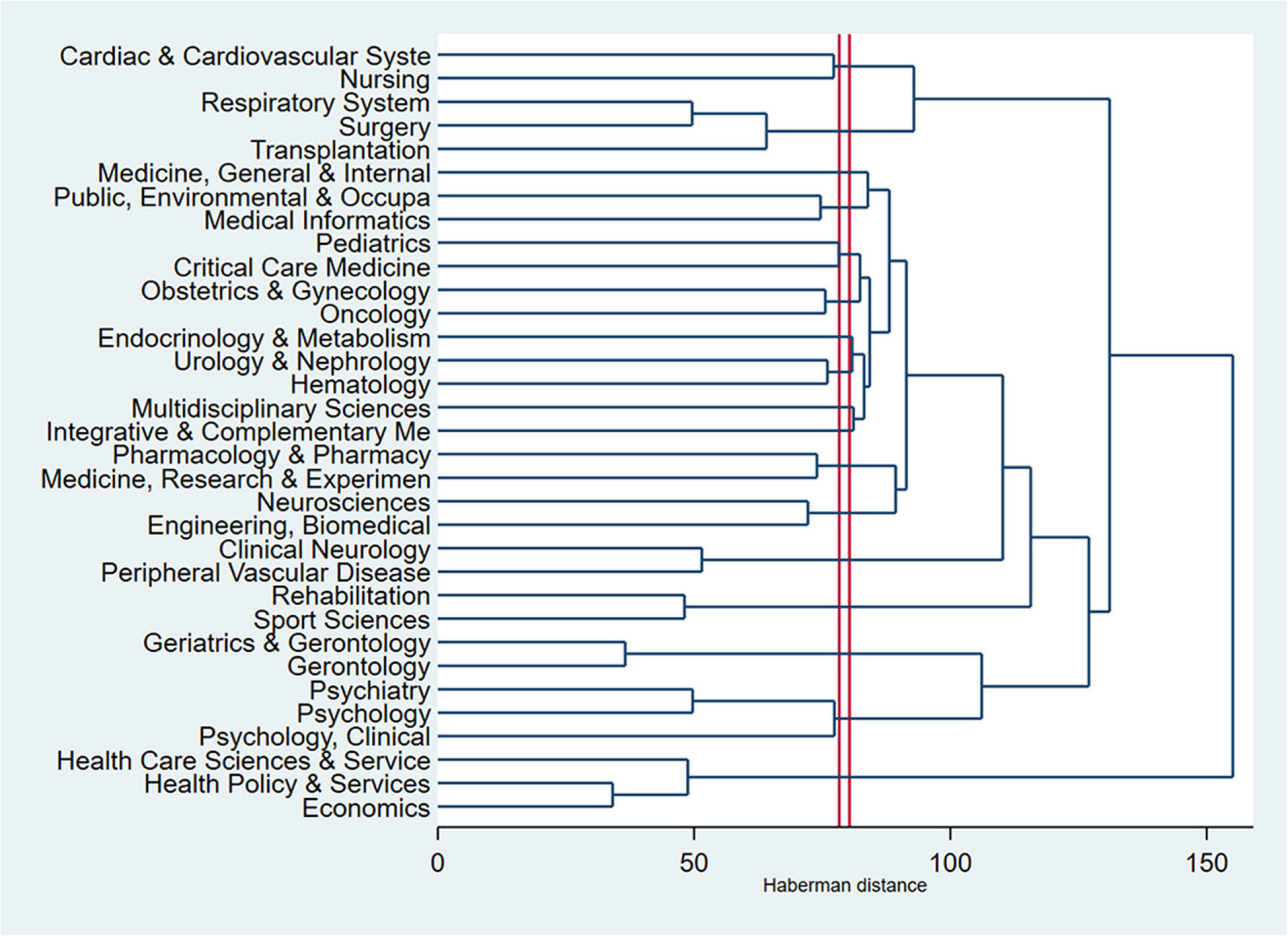
Dendrogram of coincidence of research areas using the WoS classifications

Figure 3 presents the clusters of research areas in the interventions aiming to improve QOL of CVD patients. The horizontal axis shows the distance between research areas while the vertical axis shows the research areas based on WOS categories. The research areas in these interventions could be divided into three categories 1) Cardiovascular System; 2) Prevention, Treatment (Surgery, Internal Medicine, Pharmacy and Rehabilitation); 3) Health Policy and Economic evaluation. Overall, the dendrogram indicates that health services and economics studies have not been well studied. Also, holistic (e.g., system) factors and frontline interactions with patients (e.g., nursing) seem to have influential effects on QOL of CVD patients.

## Discussion

In this study, a total of 6457 papers was analyzed to identify the global trend and the development of research landscapes in the intervention to improve QOL of CVD patients from 1990 to 2018. The number of papers increased gradually since 2002 and reached a peak in the year of 2018. By applying text mining technique based on LDA, the interdisciplinary research topics and research areas were examined in detail. The most common topics were conventional treatment (surgery and medication), and psychological, behavioral interventions.

Our study proposed a novel approach for analyzing literature that overcomes the limitations of current systematic review/meta-analysis. Indeed, the traditional systematic review and meta-analysis cannot efficiently illustrate the research development trend as this method consumes time and human resources [31]. Meanwhile, scientometrics, when standing on its own, shows the productivity, collaborations among authors, organizations, or countries and co-occurrence of author keywords, however, the approach is unable to identify the research topics underlying the current literature [32]. By combining bibliometrics and text analysis, the study was able to explore the hidden patterns of information from the literature.

This study provides an overview of interdisciplinary research landscapes in the interventions to improve QOL of CVD patients. The majority of interventions for CVDs were conventional therapies, such as surgery and medication, or psychological and behavioral interventions. Historically, the use of medications has been a primary method of CVD treatment [33], given the lower costs of drug treatment compared to hospitalization and effectiveness in relieving symptoms, which mainly aim to slow down the disease progression and improve patients’ QoL [34]. The number of patients with end-stage heart diseases as well as the introduction of the heart-lung machine and cardiopulmonary bypass leads to the increase of surgical treatment and the number of papers mentioning this therapy, which was also confirmed by other studies [35]. Besides, several associated factors to CVDs, such as obesity, smoking, and sedentary lifestyle, have been proved in previous studies [36, 37], leading to a rise in the published works regarding interventions focusing in lifestyle changes to prevent the onset of CVD episode and elevate the patients’ QOL. Notably, traditional and alternative medicine gained the concern of scientists in the last 5 years (2014–2018). This result was in the same line with previous studies [38, 39]. It might be explained that patients believed in these approaches to reduce side-effects of conventional therapy [39], yet, there has been a lack of data for the pattern use of alternative therapy for CVDs [38]. In addition, the development of technology has enabled the research of gene therapy or robot-assistant in CVD treatment [40]. However, gene therapy has shown modest success in clinical translation [41]. Meanwhile, robot-assistant has been tried and is being investigated for rehabilitation among patients after stroke [42, 43].

Findings of this study have provided several important implications for setting priority in research, designing interventions, and improving quality of care for CVD patients. Firstly, we call for interdisciplinary approaches, specifically, the integration of research areas, such as health services in providing medical care, and psycho-socio-behavioral interventions at the individual, family, health facility, and community levels. Besides, more research should be focused on the economic aspects of interventions for CVD. Finally, there has been a lack of research on interventions to reduce mental problems (e.g., depression or stress) among people with CVD; hence, future research should focus on this phenomenon since mental issues were significant risk factors for CVD [44, 45].

Our study has several limitations. First, the choice of using WOS as the only database might limit the coverage of all possible publications in interventions to improve QoL of patients with CVD compared with Google Scholar [46], or Scopus [47]. However, a previous study recommended using WoS in the case of only one available database [48]. Second, only peer-reviewed publications in the English language were included, which might have resulted in a bias against non-English publications. Furthermore, only titles and abstracts were used for content analysis, which might not provide a deep insight intro research themes of the dataset. Hence, our findings should be interpreted with caution.

## Conclusion

In conclusion, the number of scientific published works on the interventions to improve QOL among people with CVD has gradually increased from 1990 to 2018. The research areas in the field of the study emphasized the importance of interdisciplinary and inter-sectoral approaches in both evaluation and intervention. Conventional therapy (surgery and medication), and psychological and behavioral interventions were the common approach. Future research should focus on economic evaluation of intervention as well as interventions to reduce mental issues among people with CVD.

## Supplementary information

**Additional file 1.** Search query for “Quality of life” and “well-being”. Number of papers by countries as study settings

**Additional file 2.** Selection process

## Data Availability

The datasets used and analyzed during the current study are available from the corresponding author on a reasonable request.

## Abbreviations

CABG: Coronary Artery Bypass Graft Surgery
CVDs: Cardiovascular Diseases
DALY: Disability-Adjusted Life-Years
IHD: Ischemic Heart Disease
LDA: Latent Dirichlet Allocation
PCI: Percutaneous Coronary Intervention
QOL: Quality of Life
WOS: Web of Science
