# Supplementary material for: Global mapping of interventions to improve the quality of life of patients with cardiovascular diseases during 1990–2018
Source: Health Qual Life Outcomes. 2020 Jul 29;18:254. doi: 10.1186/s12955-020-01507-9 (PMC7391613; doi:10.1186/s12955-020-01507-9)
Supplement: Supplementary file 1 — Additional file 1. Search query for “Quality of life” and “well-being”. Number of papers by countries as study settings [file 12955_2020_1507_MOESM1_ESM.docx]

| No | Search query | Search result |
| --- | --- | --- |
| # 1 | TS=("quality of life") | 355,541 |
| # 2 | TS=("well-being") | 104,048 |
| # 3 | #2 OR #1 | 441,617 |
| # 4 | #2 OR #1 | 437,253 |
|  | Refined by: [excluding] Publication Years: ( 2019 ) |  |
| # 5 | #2 OR #1 | 353,171 |
|  | Refined by: [excluding] Publication Years: ( 2019 ) AND [excluding] Document Types: ( Meeting Abstract Or Proceedings Paper Or Editorial Material Or Book Chapter Or Letter Or Book Review Or Correction Or Note Or News Item Or Book Or Reprint Or Early Access Or Retracted Publication Or Biographical Item Or Correction Addition Or Discussion Or Data Paper Or Retraction Or Bibliography Or Fiction Creative Prose Or Item About An Individual Or Poetry Or Software Review ) |  |
| # 6 | #2 OR #1 | 353,170 |
|  | Refined by: [excluding] Publication Years: ( 2019 ) AND [excluding] Document Types: ( Meeting Abstract Or Proceedings Paper Or Editorial Material Or Book Chapter Or Letter Or Book Review Or Correction Or Note Or News Item Or Book Or Reprint Or Early Access Or Retracted Publication Or Biographical Item Or Correction Addition Or Discussion Or Data Paper Or Retraction Or Bibliography Or Fiction Creative Prose Or Item About An Individual Or Poetry Or Software Review ) AND [excluding] Document Types: ( Tv Review Radio Review ) |  |
| # 7 | #2 OR #1 | 327,627 |
|  | Refined by: [excluding] Publication Years: ( 2019 ) AND [excluding] Document Types: ( Meeting Abstract Or Proceedings Paper Or Editorial Material Or Book Chapter Or Letter Or Book Review Or Correction Or Note Or News Item Or Book Or Reprint Or Early Access Or Retracted Publication Or Biographical Item Or Correction Addition Or Discussion Or Data Paper Or Retraction Or Bibliography Or Fiction Creative Prose Or Item About An Individual Or Poetry Or Software Review ) AND [excluding] Document Types: ( Tv Review Radio Review ) AND [excluding]Languages: ( German Or Spanish Or French Or Portuguese Or Russian Or Turkish Or Polish Or Italian Or Korean Or Czech Or Hungarian Or Croatian Or Greek Or Dutch Or Japanese Or Slovenian Or Slovak Or Lithuanian Or Serbian Or Persian Or Malay Or Romanian Or Chinese Or Icelandic Or Arabic Or Afrikaans Or Norwegian Or Ukrainian Or Danish Or Catalan Or Swedish Or Estonian Or Bulgarian Or Serbo Croatian Or Galician Or Georgian Or Esperanto Or Finnish Or Hebrew Or Indonesian Or Welsh ) |  |
| # 8 | AU=("Anonymous" OR "anonymous") | 1,406,800 |
| # 9 | #7 NOT #8 | 327,405 |

**Additional file 1.**

**Table A1. Search query for “Quality of life” and “well-being”**

**Table A2. Number of papers by countries as study settings**

|  | **Country settings** | **Frequency** | **%** |  | **Country settings** | **Frequency** | **%** |
| --- | --- | --- | --- | --- | --- | --- | --- |
| 1 | The USA | 314 | 20.7% | 31 | Wallis and Futuna | 7 | 0.5% |
| 2 | Ireland | 150 | 9.9% | 32 | Belgium | 6 | 0.4% |
| 3 | The United Kingdom | 133 | 8.8% | 33 | Norway | 6 | 0.4% |
| 4 | Australia | 101 | 6.6% | 34 | Peru | 6 | 0.4% |
| 5 | China | 91 | 6.0% | 35 | Ghana | 5 | 0.3% |
| 6 | Canada | 56 | 3.7% | 36 | Greece | 5 | 0.3% |
| 7 | Germany | 54 | 3.6% | 37 | Malaysia | 5 | 0.3% |
| 8 | Netherlands | 53 | 3.5% | 38 | Turkey | 5 | 0.3% |
| 9 | Sweden | 44 | 2.9% | 39 | Argentina | 4 | 0.3% |
| 10 | Japan | 41 | 2.7% | 40 | Colombia | 4 | 0.3% |
| 11 | Taiwan | 34 | 2.2% | 41 | Lithuania | 4 | 0.3% |
| 12 | Oman | 33 | 2.2% | 42 | Portugal | 4 | 0.3% |
| 13 | Hong Kong | 29 | 1.9% | 43 | Serbia | 4 | 0.3% |
| 14 | New Zealand | 27 | 1.8% | 44 | Chile | 3 | 0.2% |
| 15 | Italy | 25 | 1.6% | 45 | Cuba | 3 | 0.2% |
| 16 | India | 23 | 1.5% | 46 | Mexico | 3 | 0.2% |
| 17 | Brazil | 21 | 1.4% | 47 | South Africa | 3 | 0.2% |
| 18 | Singapore | 20 | 1.3% | 48 | Czech | 2 | 0.1% |
| 19 | France | 19 | 1.3% | 49 | Egypt | 2 | 0.1% |
| 20 | Iran | 19 | 1.3% | 50 | Georgia | 2 | 0.1% |
| 21 | Spain | 19 | 1.3% | 51 | Iceland | 2 | 0.1% |
| 22 | Denmark | 17 | 1.1% | 52 | Jamaica | 2 | 0.1% |
| 23 | Finland | 16 | 1.1% | 53 | Philippines | 2 | 0.1% |
| 24 | Israel | 12 | 0.8% | 54 | United Arab Emirates | 2 | 0.1% |
| 25 | Niger | 10 | 0.7% | 55 | Antigua and Barbuda | 1 | 0.1% |
| 26 | Nigeria | 10 | 0.7% | 56 | Armenia | 1 | 0.1% |
| 27 | Poland | 10 | 0.7% | 57 | Aruba | 1 | 0.1% |
| 28 | Switzerland | 8 | 0.5% | 58 | Azerbaijan | 1 | 0.1% |
| 29 | Austria | 7 | 0.5% | 59 | Barbados | 1 | 0.1% |
| 30 | Thailand | 7 | 0.5% | 60 | Belarus | 1 | 0.1% |

Note: Table 3 shows the number of papers by the locations of study settings mentioned in the abstracts. In total, there were 1,519 papers included country settings, of those, the number of studies set up in the United States of America (USA) accounted for 20.7%. Most of the total (70%) was attributed to the top 10 countries, eight of those are developed countries. In Asia, two countries with the highest proportions of involvements as study settings, China and Japan, contributed 6.0% and 2.7% respectively.
