# Supplementary material for: Global mapping of interventions to improve the quality of life of patients with cardiovascular diseases during 1990–2018
Source: Health Qual Life Outcomes. 2020 Jul 29;18:254. doi: 10.1186/s12955-020-01507-9 (PMC7391613; doi:10.1186/s12955-020-01507-9)
Supplement: Supplementary file 2 — Additional file 2. Selection process [file 12955_2020_1507_MOESM2_ESM.docx]

**Additional file 2. Selection process**
